# Hemorrhagic diathesis in cattle due to consumption of *Adiantopsis chlorophylla* (Swartz) Fée (Pteridaceae)

**DOI:** 10.1016/j.toxcx.2020.100024

**Published:** 2020-01-23

**Authors:** Luiz Gustavo Schneider de Oliveira, Fabiana Marques Boabaid, Vaidotas Kisielius, Lars Holm Rasmussen, Florencia Buroni, Martín Lucas, Carlos Omar Schild, Fabiana López, Mizael Machado, Franklin Riet-Correa

**Affiliations:** a Instituto Nacional de Investigación Agropecuaria (INIA), Plataforma de Investigación en Salud Animal, Estación Experimental INIA Tacuarembó, Tacuarembó, Uruguay; b Polo de Desarrollo Universitario Del Instituto Superior de La Carne, Centro Universitario Regional (CENUR) Noreste, Universidad de La República, Tacuarembó, Uruguay; c Department of Technology, University College Copenhagen, Copenhagen, Denmark; d División de Laboratorios Veterinarios “Miguel C. Rubino” Regional Norte, Ministerio de Ganadería Agricultura y Pesca (MGAP), Tacuarembó, Uruguay

**Keywords:** Cattle diseases, Fern toxicity, Hematopoietic diseases, Caudatoside

## Abstract

An outbreak of acute febrile syndrome associated with coagulopathy and severe pancytopenia occurred in cattle grazing in paddocks with high infestation by *Adiantopsis chlorophylla*. The administration of the plant to a calf reproduced the same signs and lesions seen in spontaneous cases. Similar syndromes are caused by ptaquiloside from bracken fern. Traces of the ptaquiloside-like molecule caudatoside were detected together with 0.03–0.24 mg/g of it's degradation product pterosin A, in dry fronds of the plant. In conclusion, *A. chlorophylla* is a cause of hemorrhagic diathesis in cattle.

## Highlights

• *Adiantopsis chlorophylla* causes hemorrhagic diathesis in cattle.
• The main signs are acute fever, coagulopathy and severe pancytopenia.
• At necropsies, the most striking feature is a disseminated bleeding.
• Caudatoside and pterosin A are found in the plants.

## Graphical abstract

**Figure:**
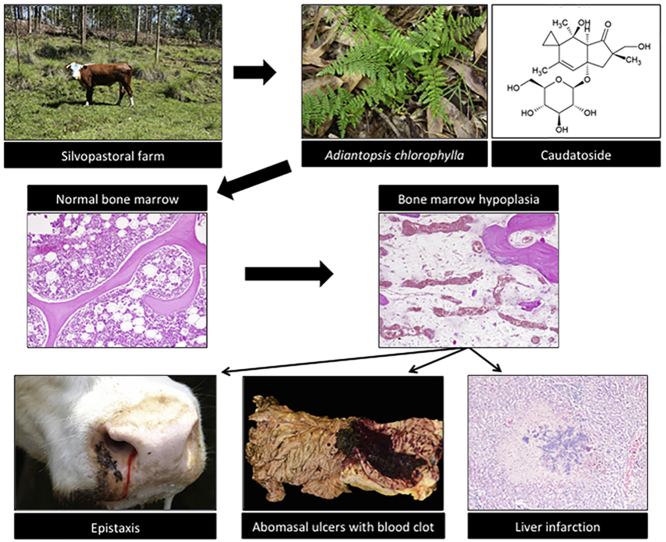

Hemorrhagic diathesis is a disease of cattle resulting from bone marrow hypoplasia, frequently associated with acute poisoning by *Pteridium* spp. In many parts of the world (França et al., 2002, Tokarnia et al., 2012, Constable et al., 2017, Boabaid et al., 2018) and by *Cheilanthes sieberi* in Australia (Clark and Dimmock, 1971). Besides the acute form, enzootic hematuria and upper alimentary tract carcinomas can result from long-term consumption of different ferns, such as the above-mentioned *Pteridium* spp. (Döbereiner et al., 1967, Tokarnia et al., 1969, Carvalho et al., 2006, Souto et al., 2006, Lucena et al., 2011), *Cheilanthes sieberi* (McKenzie, 1978, Smith et al., 1989), *Onychium contiguum* (Dawra et al., 2001), *Pteris deflexa* and *Pteris plumula* (Micheloud et al., 2017). The active principle responsible for both acute and chronic *Pteridium* spp. toxicity in ruminants is the norsesquiterpene compound ptaquiloside (Hirono et al., 1984, Yamada et al., 2007). Ptaquiloside and similar compounds from bracken and other ferns degrade naturally to pterosins. Pterosin's are found naturally in old plant material, in artificially dried samples such as fern-based food products, and are e.g. also formed in rumen juice upon digestion of ptaquiloside (Caceres-Pena et al., 2012, Gil da Costa et al., 2012, Aranha et al., 2019). Other ptaquiloside-like molecules such as caudatoside, isoptaquiloside, ptaquiloside Z, among others, are found in different species of ferns, however their toxicity is still being studied (Castillo et al., 1997, Fletcher et al., 2011). *Pteridium aquilinum* poisoning was reported in Uruguay causing enzootic hematuria in cattle in 2010 (Dutra, 2010). Outbreaks of hemorrhagic diathesis took place in Uruguay in 2019; however, the ferns involved in these cases have not been identified (Dutra, 2019). The aim of this study was to describe the occurrence of hemorrhagic diathesis in cattle associated with consumption of *Adiantopsis chlorophylla* (Pteridaceae) in northern Uruguay.

A silvopastoral farm located in the Department of Tacuarembó, in the north of Uruguay was visited to investigate an ongoing outbreak of cattle mortality during Spring 2018. Clinical inspection of the herd was done and historical data were obtained with the referring veterinarian. The herd was composed of 980 Braford, Hereford and mixed breed feeder steers (2-3 years-old), which were introduced in a forest of about 2,000ha of mature *Eucalyptus grandis* W. Hill and *Pinus elliotti* Engelm. trees in December 2017. The area was divided into paddocks of 200–300ha with a total of 100–180 cattle in each group. In addition, 30 heifers (2-3 years-old) were kept in a paddock with a natural pasture free of *Eucalyptus* and *Pinus* trees. None of the heifers were affected during the period. General sanitary measures included multivalent clostridial vaccination, and ivermectin 3.15% and closantel administration to all herd. From October to December 2018, 29 steers presented a similar clinical picture, including apathy, obtundation, muscular tremors, staggering, inappetence, hyperthermia (40–41,5 °C), drooling, epistaxis (Fig. 1A), melena or blood stained feces, sometimes containing blood clots, pale mucous membranes and poor body condition. Dyspnea, intense respiratory rasping sounds and abundant bilateral nasal mucous discharge were seen in one steer. All affected bovines died after a clinical course of three-to-seven days. Hematological exams were performed in three bovines, revealing severe thrombocytopenia (8–43 × 10^3^/mm^3^ comparing with normal 100–800 × 10^3^/mm^3^).

**Fig. 1:**
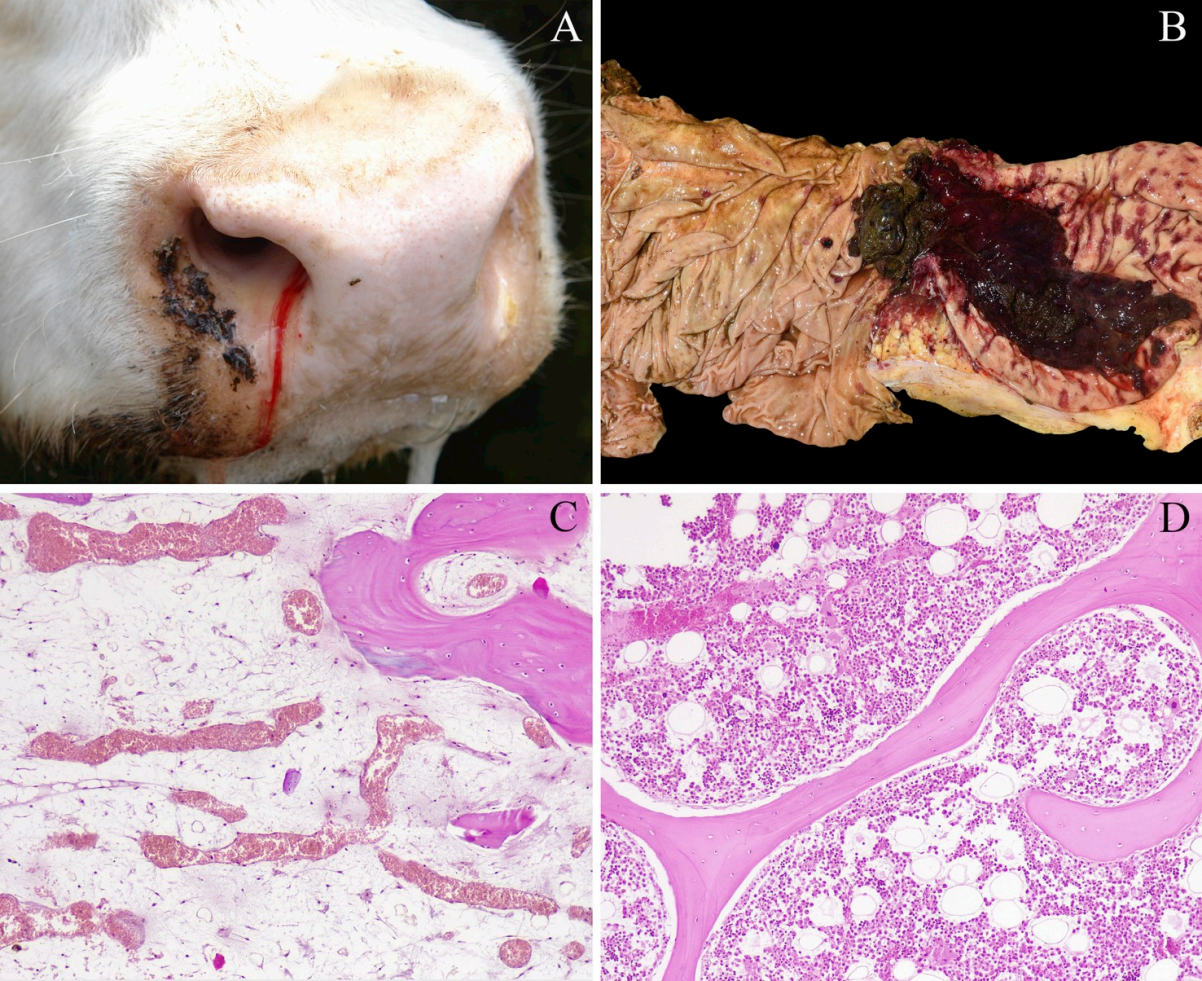
Field case of a steer with hemorrhagic diathesis presenting epistaxis and profuse salivation (A), abomasum with multifocal to coalescent ulcers with a large blood clot attached in a spontaneous case (B). Bovine with diffuse severe trilineage hypoplasia of the bone marrow (C). Bone marrow of a control steer (D).

Necropsy was performed in seven steers, which were in fair to poor body condition and exhibited pale external mucous membranes, sometimes with petechiae in ocular conjunctiva. The coat of the hindquarters was stained with blood or black feces. In some cases, blood stained feces or large blood clots were seen near the carcass. In all necropsies, the most striking feature was disseminated bleeding, including multifocal petechial to suffusive hemorrhages in subcutaneous tissue and multiple foci of hemorrhage dissecting muscular fibers. There were multifocal areas of hemorrhage in the serous surface of the rumen, omasum, abomasum, gall bladder, colon and cecum, as well as subepicardial, subendocardial, parietal peritoneum and pleura. The abomasum had multifocal to coalescing ulcers ranging from 0.2 to 10 cm in diameter in six of seven cases (Fig. 1B). The ulcers were reddened, with raised borders and sometimes had large coagula attached. Minor ulcers were seen in the rumen of four of seven steers and in the omasum of one. The colon and cecum frequently contained dark material, fibrin or blood clots. The liver of three bovines had multifocal to coalescing randomly distributed well-demarcated firm and white foci, with a reddened halo, visible on capsular and cut surfaces. In the pharynx and surrounding connective tissue of one bovine, there was moderate edema and multifocal moderate hemorrhage.

Histological examination revealed disseminated hemorrhages in different organs along with diffuse and severe reduction of all three lineages of hematopoietic cells of the bone marrow in all bovines (Fig. 1C and D). Ulceration of the abomasum (6/7 bovines), rumen (4/7) and omasum (1/7) were covered with fibrin, neutrophilic infiltrate and hemorrhage, sometimes containing multiple aggregates of coccoid to coccobacillary bacterial structures admixed. Three animals had liver infarcts with thrombosis and numerous coccoid bacterial colonies. In one case, there was expansion of the pharyngeal submucosa by moderate multifocal to coalescing edema with fibrin deposition and hemorrhage, along with multifocal thrombosis.

At field inspection all paddocks where the steers were grazing, the main weed seen in shaded fields was a species of fern, identified as *Adiantopsis chlorophylla* (Sw.) Fée (Pteridaceae), which sometimes was the only forage available (Fig. 2A–C). A voucher specimen of the plant is deposited at the herbarium of the Agronomy Faculty of the University of the Republic of Uruguay with the accession number MVFA 34989. Plants pertaining to the genus *Pteridium* were not found and other species of ferns, such as *Blechnum australe* ssp. *auriculatum* (Cav.) Sota, *Thelypteris* sp. and *Doryopteris concolor* (Langsd. & Fisch.) Kuhn & Decken were only seen in minimal amount.

**Fig. 2:**
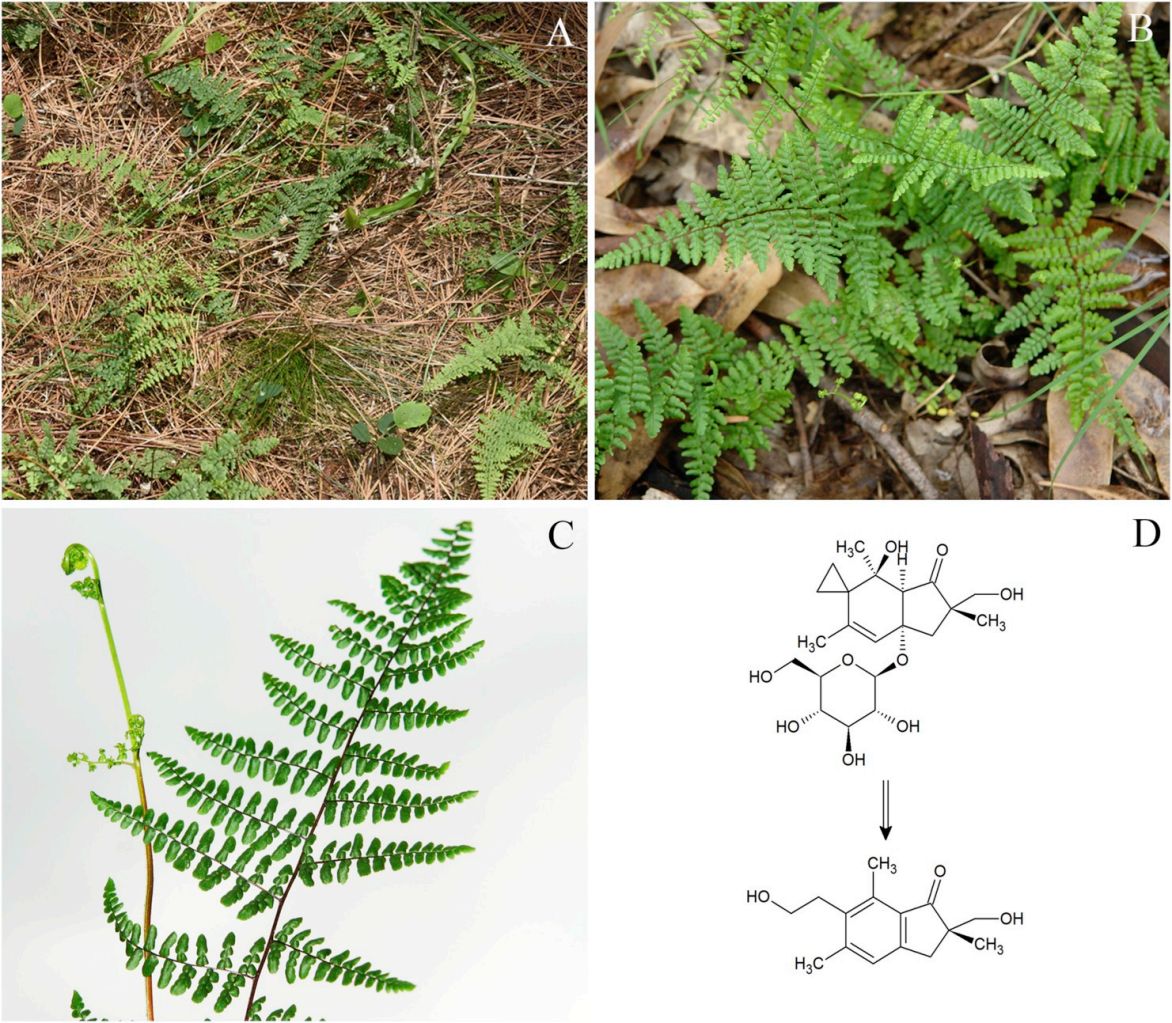
Paddock with high infestation by *Adiantopsis chlorophylla* in a *Pinus elliotti* forest (A), and a closer view of the plant in a *Eucalyptus grandis* forest (B). Adaxial surface of a frond, besides a fiddlehead of *A. chlorophylla* (C). Formation of pterosin A from caudatoside (gross reaction) (D).

Based on the clinical and pathological evidence of bovine hemorrhagic diathesis and on the abundance and ubiquity of *A. chlorophylla* in the farm, an experiment was carried out in order to determine the toxicity of the plant for bovine according to the National Commission of Animal Experimentation (CNEA – Uruguay) practices (protocol number INIA, 2018.14). Two 1-year-old Braford calves with similar body weight (142 kg case and 147 kg control) were used. One of the calves ingested a daily dose of 22.5  g/kg BW of fresh fronds of *A. chlorophylla* and the other calf was kept as a control. At the 21st day, the experimental calf developed the first signs, after the ingestion of a total dose of 67.1 kg of fresh plant material.

Clinical signs started with hyperthermia, inappetence, prostration weakness, and drooling. At the third day, the mucous membranes were pale with multifocal petechial hemorrhages. Uncontrollable bleeding after skin or venous puncture, and hindquarter coat stained with black feces were noted. The calf presented also teeth grinding and respiratory rasping sounds. At the final stage, the calf was in a poor body condition, presented unstable gait and assumed sternal recumbence. Six days after the first signs the calf died. Hematological exams revealed severe thrombocytopenia (21 × 10^3^/mm^3^) in a sample obtained the day before death.

At necropsy, the calf exhibited disseminated hemorrhages in the subcutaneous tissue. The abdominal cavity had moderate deposition of fibrin. Multifocal petechial and/or suffusive hemorrhages were seen in different organs, especially in the spleen, gall bladder and the left ventricular endocardium. The pericardial sac presented moderate fibrin deposition. The retropharyngeal connective tissue was edematous and the local lymph nodes were enlarged and had multifocal well-demarcated hemorrhagic areas on cut surface. The abomasum and rumen had multifocal erosions and ulcers ranging from 0.1 to 3 cm. The colon and cecum had moderately thickened walls, with hemorrhagic contents and fibrin clots and the cecum had multifocal to coalescent ulcers measuring 0.1–2 cm. The jejunum had a focal well-delimited hemorrhagic area with 2 cm elevating the serosal surface.

Histologically, there was multifocal hemorrhages and thrombosis in different organs. The visceral and parietal peritoneum, especially the liver capsule, the gall bladder and the intestinal serosa were covered with moderate to large amounts of fibrin, sometimes containing myriads of coccobacillary structures (bacterial colonies). The liver had also miliary foci of well-delimited coagulative necrosis in the parenchyma (infarcts), associated with myriads of coccoid to bacillary structures and multifocal thrombosis. The abomasum, rumen and cecum had multifocal ulcers with multifocal thrombosis and myriads of coccobacillary bacteria. The jejunum had a focally extensive hemorrhagic infarct with multifocal thrombosis. The pharyngeal mucosa had a focally extensive ulcer and the submucosa was expanded by moderate edema with abundant fibrin and myriads of coccobacillary bacteria. The palatine tonsils and the retropharyngeal lymph nodes had multifocal infarcts and multifocal thrombosis, with diffuse severe hemorrhage and edema and myriads of coccobacillary bacteria intermingled. The bone marrow had a severe diffuse depletion of the three hematopoietic lineages. The control calf showed no clinical signs and the hematological parameters were within normal range.

Detection and quantification of the norsesquiterpene glycosides ptaquiloside, ptesculentoside and caudatoside as well as their corresponding pterosin's B, G and A was performed on LC-MS (Agilent 1260 Infinity HPLC System; Agilent 6130 Single Quadropol (Kisielius et al., 2020). *Adiantopsis chlorophylla* fronds were collected at the paddocks where the affected cattle were grazing and dried for 40 days under natural conditions. A total of six samples were collected in different parts of the affected fields (approx. 40 g fresh weight each). The leaves and steams were shipped to Denmark, grinded to a fine powder and kept for 5 weeks at −20 °C until analysis at University College Copenhagen. Traces of caudatoside were found in 5 out of 6 samples. In addition, pterosin A originating from caudatoside in the fresh material was detected in all samples (0.03–0.24 mg/g). No ptaquiloside nor ptesculentoside or associated pterosins were detected.

Clinical signs and hematological features, as well as necropsy and histological findings were compatible with hemorrhagic diathesis both in spontaneous field and in experimental cases. Our cases of *A. chlorophylla* toxicity are characterized by a bleeding disorder secondary to bone marrow trilineage depression, similar to what is described in acute bracken fern poisoning (Anjos et al., 2008).

Coagulopathy results from decreased production of platelets, while fever and liver infarcts can be attributed to the opportunistic infections due to the overwhelming neutropenia, both secondary to bone marrow lesion (Naftalin and Cushnie, 1954, Anjos et al., 2008, Constable et al., 2017). Another prominent finding is multifocal ulceration in the abomasum, frequently associated with massive gastrointestinal bleeding. Some authors propose that gastrointestinal ulceration could be a consequence of the high humoral histamine levels found in acute bracken toxicosis. Similarly, in cases with respiratory signs, laryngeal edema is attributed to mast cell degranulation (Constable et al., 2017). In our experimental case, besides laryngeal and pharyngeal edema and hemorrhage, there were multifocal infarcts in the palatine tonsils and retropharyngeal lymph nodes, which could have accentuated the respiratory sounds.

The disease is classically related to ptaquiloside containing plants, represented by bracken and other ferns. Ptaquiloside is responsible for both acute and chronic forms of toxicity (Yamada et al., 2007, Gil da Costa et al., 2012). The molecule found in the *A. chlorophylla* population involved in the outbreak reported here was identified as caudatoside, an illudane-type β-glucoside isolated for the first time from *Pteridium caudatum* (L.) Maxon from Venezuela (Castillo et al., 1997). As far as we know, this ptaquiloside-like compound has never solely been related to spontaneous or experimental cases of hemorrhagic diathesis in ruminants before. The large contents of pterosin A in our *A. chlorophylla* samples is probably a result of the hydrolysis of the caudatoside due to sample handling and may correspond to a caudatoside content of up to approx. 0,4 mg/g (assuming a molar conversion ratio of 1:1 and a molar ratio of 1.7; Caceres-Pena et al., 2012, Gil da Costa et al., 2012, Fletcher et al., 2011, Rasmussen and Pedersen, 2017)).

*Adinatopsis chlorophylla* (Pteridaceae) is found in Central and South America, ranging from Mexico to northern Argentina and southern Brazil. It is found in humid areas with different degrees of disturbance and was appointed as an invasive species in the pampean grasslands of Argentina. The presence of allelopathic and/or cyanogenic substances was tentatively attributed to the fern, since herbivorism inhibition could explain the increasing predominance of this plant in the pampean rangelands (Boccanelli and Pire, 2011).

In our study, *A. chlorophylla* was seen largely associated with shaded paddocks, in mature *Eucalyptus* and *Pinus* stands, where the fern was the predominant species of weed at the forest floor. An association between tree stands and the distribution of *A. chlorophylla* deserves more investigation, as the area occupied by silviculture increased substantially in the last decades in Uruguay (DIEA-MGAP, 2018). Recent reports of similar outbreaks of hemorrhagic diathesis in cattle in silvipastoral systems in the neighboring Cerro Largo Department, besides anecdotal evidences of the disease in other parts of the Country raise concerns that the condition could be spreading with the ecological changes produced by forestation (Dutra, 2019). Moreover, there are still species of ferns with undetermined contents of ptaquiloside, caudatoside or similar compounds in South America, which could represent a risk for livestock and for humans. In this outbreak the toxicity and/or the presence of norsesquiterpene glycosides in other species of ferns found in small amounts in the paddock was not analyzed, thus their participation in the etiology of the disease cannot be ruled out. Recently, two species of ferns, *Pteris deflexa* and *Pteris plumula* were reported to cause bovine enzootic hematuria in northwestern Argentina (Micheloud et al., 2017).

Based on 1) Epidemiological evidence, clinical signs and pathological findings on spontaneous cases; 2) Detection of caudatoside in the plant, and 3) Experimental reproduction of the disease in a bovine, we conclude that *Adiantopsis chlorophylla* is a cause of hemorrhagic diathesis in cattle.

## CRediT authorship contribution statement

**Luiz Gustavo Schneider de Oliveira:** Data curation, Writing - original draft, Writing - review & editing. **Fabiana Marques Boabaid:** Data curation, Writing - original draft, Writing - review & editing. **Vaidotas Kisielius:** Writing - review & editing, Formal analysis. **Lars Holm Rasmussen:** Writing - review & editing, Formal analysis. **Florencia Buroni:** Data curation, Writing - review & editing. **Martín Lucas:** Data curation, Writing - review & editing. **Carlos Omar Schild:** Data curation, Writing - review & editing. **Fabiana López:** Data curation, Writing - review & editing. **Mizael Machado:** Data curation, Writing - review & editing. **Franklin Riet-Correa:** Data curation, Writing - original draft, Writing - review & editing.

## Declaration of competing interest

On behalf of all the authors, the corresponding author states that there is no conflict of interest.
